# Electrophysiological, Morphological, and Ultrastructural Features of the Injured Spinal Cord Tissue after Transplantation of Human Umbilical Cord Blood Mononuclear Cells Genetically Modified with the VEGF and GDNF Genes

**DOI:** 10.1155/2017/9857918

**Published:** 2017-03-21

**Authors:** Y. O. Mukhamedshina, Z. E. Gilazieva, S. S. Arkhipova, L. R. Galieva, E. E. Garanina, A. A. Shulman, G. G. Yafarova, Y. A. Chelyshev, N. V. Shamsutdinova, A. A. Rizvanov

**Affiliations:** ^1^Kazan Federal University, Kazan, Russia; ^2^Kazan State Medical University, Kazan, Russia; ^3^Republic Clinical Hospital, Kazan, Russia; ^4^Kazan State Academy of Veterinary Medicine, Kazan, Russia

## Abstract

In this study, we examined the efficacy of human umbilical cord blood mononuclear cells (hUCB-MCs), genetically modified with the VEGF and GDNF genes using adenoviral vectors, on posttraumatic regeneration after transplantation into the site of spinal cord injury (SCI) in rats. Thirty days after SCI, followed by transplantation of nontransduced hUCB-MCs, we observed an improvement in H (latency period, LP) and M(*A*_max_) waves, compared to the group without therapy after SCI. For genetically modified hUCB-MCs, there was improvement in *A*_max_ of M wave and LP of both the M and H waves. The ratio between *A*_max_ of the H and M waves (H_max_/M_max_) demonstrated that transplantation into the area of SCI of genetically modified hUCB-MCs was more effective than nontransduced hUCB-MCs. Spared tissue and myelinated fibers were increased at day 30 after SCI and transplantation of hUCB-MCs in the lateral and ventral funiculi 2.5 mm from the lesion epicenter. Transplantation of hUCB-MCs genetically modified with the VEGF and GNDF genes significantly increased the number of spared myelinated fibers (22-fold, *P* > 0.01) in the main corticospinal tract compared to the nontransduced ones. HNA^+^ cells with the morphology of phagocytes and microglia-like cells were found as compact clusters or cell bridges within the traumatic cavities that were lined by GFAP^+^ host astrocytes. Our results show that hUCB-MCs transplanted into the site of SCI improved regeneration and that hUCB-MCs genetically modified with the VEGF and GNDF genes were more effective than nontransduced hUCB-MCs.

## 1. Introduction

The use of gene and cell therapy is proving to be a promising strategy for overcoming the consequences of spinal cord injury (SCI). Recent studies have shown that a combination of both types of therapy is the most effective tool for SCI treatment [1, 2]. An active field of study is genetically modified stem and progenitor cells that are capable of providing enhanced expression of neuroprotective genes and stimulation of regeneration at the site of injury. The promise of this approach includes (1) the introduction of therapeutic genes into stem cells to provide beneficial effects [3]; (2) the ability of grafted cells to replace dead spinal cord cells by neurogenic differentiation, including effects of introduced genes [1, 4, 5]; and (3) secretion of proteins encoded by transgenes that may have antiapoptotic and neuroprotective effects on CNS cells, stimulate attachment of the growing axon to the substrate (via adhesion molecules), and maintain axon elongation, myelin formation, and remyelination.

Human umbilical cord blood mononuclear cells (hUCB-MCs), a well-known source of stem and progenitor cells [6], are promising both as regenerative cells and as a therapeutic gene carrier for the treatment of traumatic SCI. Transplantation into the SCI of genetically modified by NT-3 gene human mesenchymal stem cells derived from umbilical cord blood has been demonstrated to reduce pathological cavitation, confer protection to myelinated fibers, and promote functional recovery [7]. Previously, we determined that genetic modification of hUCB-MCs with* vegf*,* fgf2,* and* gdnf* genes, when transplanted into the SCI area in rats, enhanced the therapeutic effect of these cells [8–10]. Analysis of these data suggested that the gene-cell hUCB-MC construct with a combination of the* vegf* and* gdnf* genes had the greatest potential to enhance posttraumatic recovery and improve the regenerative impact of the carrier cells. Improvement in outcomes associated with overexpression of GDNF and VEGF in SCI may be mediated by (1) a supporting influence of these factors on survival of motor and sensory neurons by curbing apoptosis [11–15]; (2) stimulation of neurogenesis and axon growth [16–18]; and (3) enhancement of neovascularization and proliferation of neural stem cells and Schwann cells [19–22]. Electrophysiological and electron microscopic analyses were used to investigate the impact of the constructs on the structure and function of the injured spinal cord. We evaluated the neuroregenerative ability conferred by hUCB-MCs in injured rat spinal cord, as well as their influence on phenotype of engrafted cells when genetically modified with the recombinant adenoviruses encoding VEGF and GDNF. Such combination of VEGF and GDNF had not been previously investigated.

## 2. Materials and Methods

### 2.1. hUCB-MCs

hUCB was obtained from normal full-term pregnant women in accordance with the Protocol and Standards of the Stem Cell Bank of Kazan State Medical University. The study was approved by the Institutional Review Board of Kazan State Medical University (Kazan, Russian Federation). Written informed consent was obtained from all pregnant mothers according to the clinical and experimental research protocol, approved by the Local Ethic Expert Committee of the Kazan State Medical University (number 195, 10 May 2010). hUCB-MCs were isolated and transduced by adenoviral vectors encoding VEGF and GDNF as previously described [23].

### 2.2. Animals and Spinal Cord Injury

All animal protocols were approved by the Kazan Federal University Animal Care and Use Committee (Permit Number: 5 dated 27 May 2014). Twenty-nine adult male and female Wistar rats (weight, 250–300 g each; Pushchino Laboratory, Russia) were group-housed in clear plastic cages (12 h: 12 h light/dark cycle) with food and water available ad libitum.

Anesthesia and surgical techniques were those described previously [23]. Briefly, rats were deeply anesthetized with chloral hydrate (80 mg/mL, 0.4 mL per 100 g, Sigma-Aldrich). After skin incision, the Th8 vertebral level was removed by laminectomy. The impact rod (diameter 2 mm, 10 g) of an impactor was centered above Th8 and dropped from a height of 25 mm to induce SCI [8]. After SCI, cells were injected in the hUCB-MC groups; then the dorsal back musculature and skin were sutured. Following surgery rats received doses of gentamicin (25 mg/kg, Omela, Russian Federation) intramuscularly for 7 consecutive days. Bladders of injured rats were manually emptied twice daily until spontaneous voiding occurred.

### 2.3. Cell Transplantation

Rats were assigned randomly to four groups: (1) hUCB-MCs transduced with recombinant adenoviral vectors encoding VEGF and GDNF injected after SCI (SCI hUCB-MCs + Ad5-VEGF + Ad5-GDNF, *n* = 8), (2) nontransduced hUCB-MCs injected after SCI (SCI hUCB-MCs, *n* = 8), (3) no injection after SCI (SCI, *n* = 8), and (4) no SCI (intact, *n* = 5). For the two hUCB-MC groups intraspinal injections were at two points (5 *μ*L/injection; 1 × 10^6^ cells/5 *μ*L Dulbecco's phosphate-buffered saline) after SCI as described previously [9]. Thirty days after SCI rats were anesthetized with chloral hydrate and intracardiacally perfused with 4% paraformaldehyde (PFA, Sigma-Aldrich) in phosphate-buffered saline (PBS, pH 7.4) for routine histology and 4% PFA with 0.2% glutaraldehyde in PBS for evaluation of spared myelinated fibers and electron microscopy (4°С).

### 2.4. Electrophysiological Evaluation

Electrophysiological tests were performed in intact rats and all other rats 30 days after SCI. Rat neuromotor function was assessed by stimulating electromyography. Needle electrodes were used to record the direct muscle response (M wave) and the monosynaptic reflex response (H wave) from the gastrocnemius muscle in response to electrical stimulation of the tibial nerve (right and left). Stimulating electrodes were inserted into the projection area of the tibial nerve in the popliteal fossa (right and left). Intensity and duration of stimulus were varied from 0.35 B to 60 B and 0.5 ms, respectively. We analyzed the following of H and M wave parameters: threshold, latency period (LP), maximum amplitude of the responses (*A*_max_), and the ratio of H wave *A*_max_ and M wave *A*_max_ (H_max_/M_max_), expressed as a percentage. Values for the hind limbs of each rat were averaged.

### 2.5. Morphometric Analysis

After initial fixation, cord tissue centered on the injury epicenter for the SCI groups or Th8 vertebral level of intact rats was fixed in 2.5% glutaraldehyde in PBS for 24 h at 4°С. After postfixation and staining with 1% osmium tetroxide for 2 h, and dehydration, pieces of spinal cord were embedded in Epon. 1 *μ*m transverse tissue sections obtained using an Ultracut Leica EM UC7 (Leica EM, Germany) were stained with methylene blue to visualize myelinated fibers. Images were captured using a ×100 objective lens with an Axio Lab.A1 microscope (Carl Zeiss) equipped with a digital camera (AxioCam HRc, Carl Zeiss). Spared myelinated fibers in the main corticospinal tract (CST) and the lateral and ventral funiculi (LF and VF, S = 0.01 mm^2^) were counted on both sides in spinal segments 2.5 mm rostral and caudal to the injury site [10]. ImageTool application version 3.0 was used for measuring the spared myelinated fibers. Some longitudinal sections were stained with azure-eosin stain or Weigert-Van Gieson's stain to visualize elastic fibers and collagen.

### 2.6. Electron and Immuno-Electron Microscopy

For electron microscopy, ultrathin sections (transverse and longitudinal) mounted on copper grids (Sigma-Aldrich, USA, 200 mesh) were incubated with uranyl acetate and lead citrate for double contrast. Sections were examined with a transmission electron microscope, Jeol 1200 SX (Tokyo, Japan). For immunoelectron microscopy, after dehydration, pieces of spinal cord were embedded in LR-white (Sigma, USA). Ultrathin sections were mounted on nickel grids with formvar film (Sigma-Aldrich, USA, 200 mesh) and blocked with TBS-NGS-BSA-Tx100 (Tris-buffered saline (Tris 0.01 M, NaCl 0.15 M pH = 8.2), normal goat serum 10%, bovine serum albumin 0.2%, and Triton X-100 0.1%) for 1 h. After rinsing in TBS, sections were incubated overnight at 4°C with anti-glial fibrillary acidic protein (GFAP, Santa Cruz, 1 : 150) and anti-human nuclei antigen (HNA, Millipore, 1 : 500) antibodies (Ab) and then 10 nm gold-conjugated secondary Ab (Sigma-Aldrich, USA) for 1 hour at room temperature. For enhanced visualization, silver (Silver Enhancer Kit, Sigma-Aldrich, USA) was deposited on the colloidal gold. Then, sections were double-contrasted with uranyl acetate for 20 min at 60°C and lead citrate for 10 min at room temperature; they were examined by transmission electron microscopy.

### 2.7. Statistical Analysis

Data are presented as means ± standard error of the mean (SEM). Student's* t-*test, a one-way analysis of variance (ANOVA) with Tukey's test, or two-way analysis of variance (ANOVA) was used for multiple groups. Values of *P* < 0.05 and *P* < 0.01 were considered statistically significant. Data were analyzed using Origin 7.0 SR0 Software (OriginLab, Northampton, MA, USA).

## 3. Results

### 3.1. Electrophysiology Results Indicate That Transplantation into the Area of SCI of Genetically Modified hUCB-MCs Was More Effective Than Nontransduced hUCB-MCs

**Figure 1:**
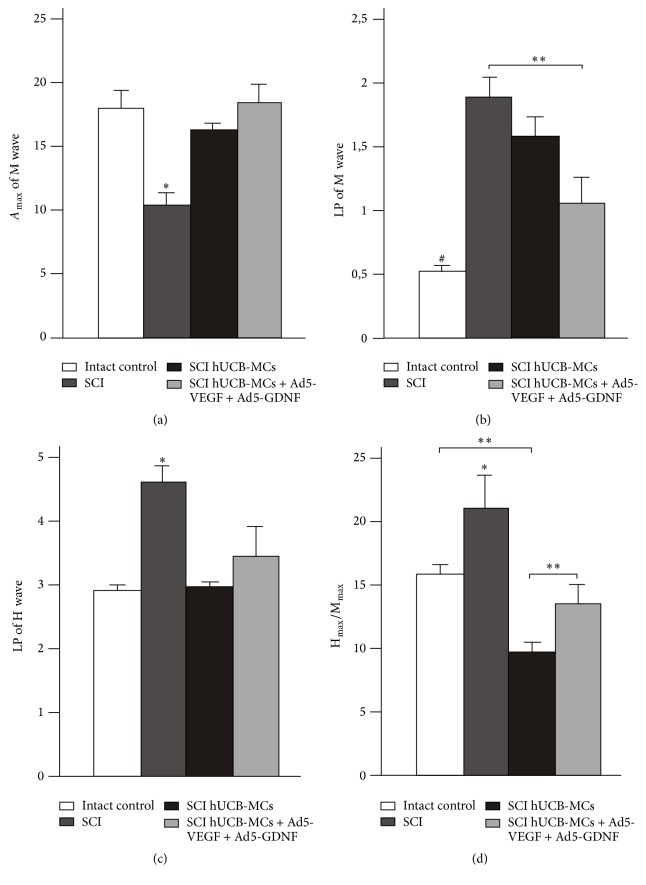
Electrophysiology results. *A*_max_ of M wave (a), LP of M and H wave ((b) and (c), resp.), and the ratio between H and M wave amplitudes (d) after spinal cord contusion in experimental groups. Differences were statistically significant between SCI and other experimental groups (^*∗*^*P* < 0.05). Differences were also statistically significant between intact and other experimental groups (^#^*P* < 0.01). ^*∗∗*^*P* < 0.05, one-way ANOVA followed by a Tukey's post hoc test.

Thirty days after SCI, *A*_max_ of the M wave was significantly decreased, by 40%, in the SCI group, compared to the intact group (Figure 1(a)) (10.31 ± 1.7 in the SCI versus 17.04 ± 1.9 in intact group, *P* < 0.05, *n* = 8 in both groups). *A*_max_ of the M wave did not change significantly in SCI hUCB-MCs and SCI hUCB-MCs + Ad5-VEGF + Ad5-GDNF groups, compared to intact group. By day 30, SCI rats made multiple attempts at behaviors such as extensive movement of two-three joints of the hind limb. SCI hUCB-MCs rats had intermediate recovery and showed occasional or frequent to consistent weight supported plantar steps with no forelimb-hind limb coordination, whereas SCI hUCB-MCs + Ad5-VEGF + Ad5-GDNF rats had a late phase of recovery and showed consistent plantar stepping and forelimb-hind limb coordination.

Previously, Brouwer et al. reported that a significant reduction in the conduction velocity in the peripheral nerve was not observed after SCI [24]. However, in our experiments, the LP of the M wave, which characterizes the speed of conduction of the motor fibers in the mixed nerves, increased in all experimental groups compared to the intact group (1.05 ± 0.4 in SCI hUCB-MCs + Ad5-VEGF + Ad5-GDNF; 1.61 ± 1.25 in SCI hUCB-MCs; and 1.9 ± 0.17 in SCI versus 0.53 ± 0.1 in intact group, *P* < 0.05, *n* = 8 in both groups). At day 30, the maximum reduction of the speed of conduction was observed in SCI rats (average about 3.5-fold) (Figure 1(b)). The LP of the M wave in SCI hUCB-MCs + Ad5-VEGF + Ad5-GDNF rats was closer to the value in intact rats.

In SCI rats, we observed that the LP of the H wave had a significant increase, 65%, compared to intact group (Figure 1(c)) (4.61 ± 0.33 in SCI versus 2.97 ± 0.3 in intact group, *P* < 0.05, *n* = 8 in both groups), thus indicating a development in delay of conduction along the arc of the H-reflex. Differences were not statistically significant between intact group and hUCB-MC injected groups (2.99 ± 0.3 in SCI hUCB-MCs versus 2.97 ± 0.3 in intact group, *n* = 8 in both groups). Amplitude of the H-reflex had a large variability. As a result, the ratio between *A*_max_ of the H wave and *A*_max_ of the M wave (H_max_/M_max_) is an important parameter that reflects the true value of the H-reflex. 30 days after SCI, the H_max_/M_max_ ratio had a significant increase, 31%, in SCI group, compared to intact group (Figure 1(d)) (21.2 ± 2.7 in the SCI versus 16.41 ± 0.85 in intact group, *P* < 0.05, *n* = 8 in both groups). The ratio in intact animals was similar (no significant difference) to the group with injection of hUCB-MCs + Ad5-VEGF + Ad5-GDNF after the injury (16.41 ± 0.85 in intact group versus 13.77 ± 3.5 in SCI hUCB-MCs + Ad5-VEGF + Ad5-GDNF, *n* = 8 in both groups). Interestingly, the H_max_/M_max_ ratio was lower in the SCI hUCB-MCs group (9.54 ± 1.38) compared to the intact and other groups (*P* < 0.05).

### 3.2. Genetically Modified hUCB-MCs Reversed the Degeneration of Myelinated Fibers in the White Matter of Contused Rat Spinal Cord

**Figure 2:**
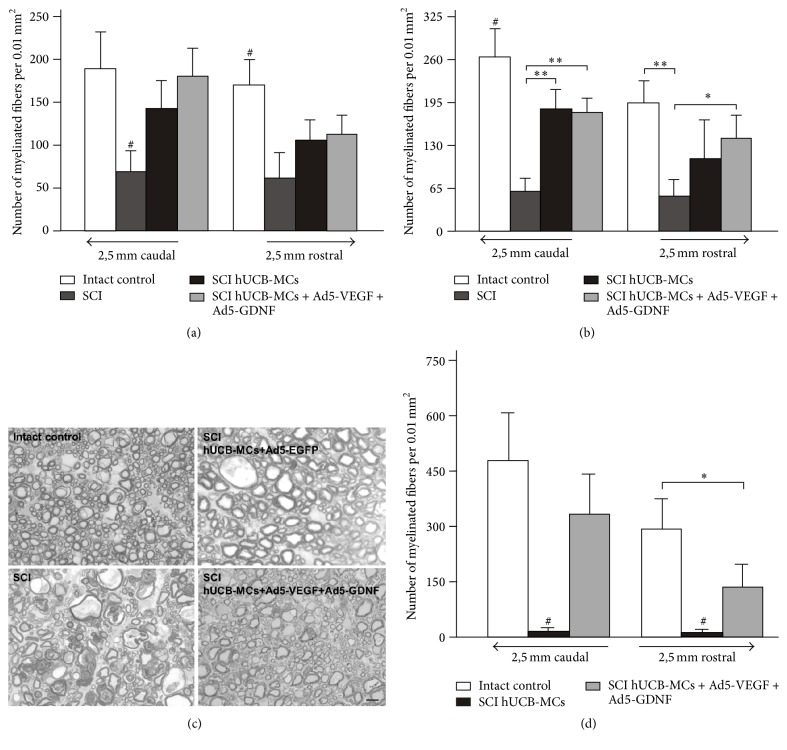
Analysis of myelinated fibers. The average number of spared myelinated fibers in the lateral (a) and ventral funiculi (b) of spinal segments 2.5 mm rostral and caudal to the lesion epicenter at day 30 after injury. Differences were statistically significant between SCI/intact and other experimental groups (^#^*P* < 0.05). ^*∗*^*P* < 0.05, ^*∗∗*^*P* < 0.01 one-way ANOVA followed by a Tukey's post hoc test. (с) Fragments of the ventral funiculi at a distance of 2.5 mm from the SCI/Th8 epicenter in the caudal direction at day 30 after injury. The images are methylene blue-stained semithin sections. Scale bar: 10 *μ*m. (d) The average number of spared myelinated fibers in the CST of spinal segments 2.5 mm caudal and rostral to the lesion epicenter/Th8 at day 30 after injury. Differences were statistically significant between SCI hUCB-MCs and other experimental groups (^#^*P* < 0.01). ^*∗*^*P* < 0.05 one-way ANOVA followed by a Tukey's post hoc test.

At 30 days after SCI we observed no difference in the number of spared myelinated fibers in VF and LF between groups injected with hUCB-MCs (unmodified or genetically modified) (Figure 2). The preservation of myelinated fibers in these areas was lower after injection of hUCB-MCs in the lesion site of the spinal cord compared to intact spinal cord. However, no differences were detected between the intact, SCI hUCB-MCs, and SCI hUCB-MCs + Ad5-VEGF + Ad5-GDNF groups in the VF and LF at a distance of 2.5 mm from the epicenter in either the caudal or rostral direction (Figures 2(a) and 2(b)). In the SCI group, the number of spared myelinated fibers was lower in all areas studied compared to other groups.

Preservation of myelinated fibers in CST at a distance of 2.5 mm from the epicenter in both directions was analyzed. The number of spared myelinated fibers was minimal (average 12–15 per 0.01 mm^2^) in the SCI hUCB-MCs group and absent in the SCI group (Figures 2(c) and 2(d)). In the SCI hUCB-MCs + Ad5-VEGF + Ad5-GDNF group, the number of spared myelinated fibers was higher relative to SCI hUCB-MCs group at 2.5 mm from the epicenter in both rostral (11.7-fold, *P* < 0.01) and caudal (22-fold, *P* < 0.01) directions. In contrast, no differences were detected between the intact and SCI hUCB-MCs + Ad5-VEGF + Ad5-GDNF groups at a distance of 2.5 mm from the epicenter in the caudal direction (470.4 ± 253 in intact versus 331 ± 121.3 in SCI hUCB-MCs + Ad5-VEGF + Ad5-GDNF, *n* = 8 in both groups). Thus, adenoviral vector-mediated gene transfer of VEGF and GDNF reversed the degeneration of myelinated fibers in the CST of contused rat spinal cord. However, unmodified or genetically modified hUCB-MCs exerted the same positive influence on the preservation of myelinated fibers in VF and LF at 2.5 mm from the injury epicenter.

### 3.3. Electron Microscopic Analysis

#### 3.3.1. The Morphological and Ultrastructural Features of the Spinal Cord Tissue at Day 30 after SCI and Transplantation of hUCB-MCs

**Figure 3:**
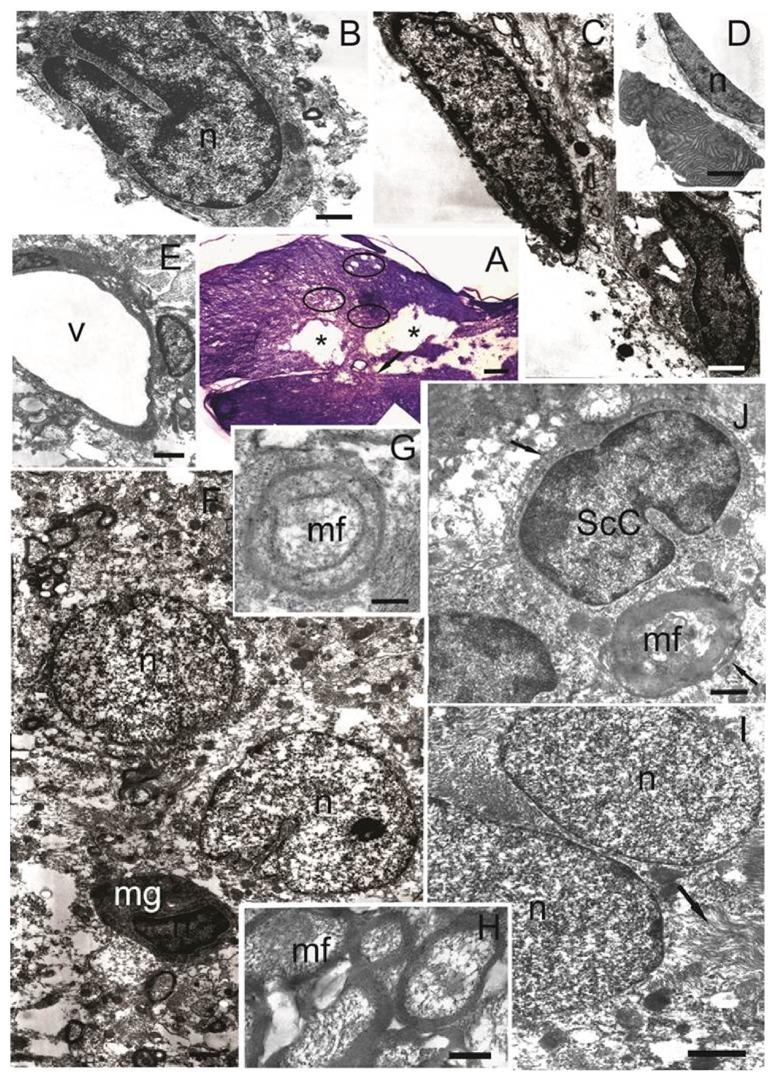
The morphological and ultrastructural features of the spinal cord tissue at day 30 after SCI and transplantation of hUCB-MCs. (А) Longitudinal section of the spinal cord (Weigert-Van Gieson's stain). Asterisks show posttraumatic cavities, connected by narrow bands of cells. Arrow shows red-violet precipitate, indicating a specific reaction to the connective tissue. Marked oval areas indicate fields with small posttraumatic cavities. (B) Cell with irregularly or lobed-shaped nuclei containing heterochromatin in which lysosomes are visualized in the cytoplasm. Plasma membrane of this cell forms narrow invaginations that surround the remnants of degenerating nerve elements. (C) Two types of cells are located on the edge of posttraumatic cavity. Astrocytes have an elongated shape with electron-dense cytoplasm and elongated nucleus with euchromatin high electron density. Macrophages also have an elongated shape and electron-dense cytoplasm with lysosomes. The nucleus has an elongated irregular shape with heterochromatin. (D) Narrow spindle-shaped cell lines the edge of posttraumatic cavity and has an elongated shape nucleus with electron-dense cytoplasm. Organelles in the cytoplasm of cell are poorly identified. Near some of these cells we observed apoptotic bodies, consisting of electron-dense concentric lamellar structures along the periphery. (E) Blood capillary in the cavity border. (F) Two types of cells located in the cavity border. Astrocytes have electron-transparent cytoplasm with electron-dense small sized mitochondria and processes extending between the nerve elements and vessels. Microglia are small cells with electron-dense nucleus and cytoplasm in which cisternae of endoplasmic reticulum have a lighter intermembrane space. (G) Mesaxon myelination on the axial cylinder. (H) Clusters of small myelinated fibers with a very thin myelin sheath in the rostral directions from the epicenter of injury. (J) Myelin-forming Schwann cells. Arrows indicate the basal membrane. (I) Fibrous astrocytes have cytoplasm with a plurality of parallel extending bundles of intermediate filaments (arrow). Scale bar: 200 *μ*м (А), 1 *μ*м (B, C, G), 2 *μ*м (H, I), 5 *μ*м (D, F), 7 *μ*м (E), and 500 nm (J). n: nucleus; v: vessel; mf: myelin fiber; ScC: Schwann cell; mg: microglial cell.

Thirty days after SCI and transplantation of hUCB-MCs we observed a traumatic centromedullary cavity into which bands of cells sprouted (Figure 3(А)). In this cavity we identified cells with irregular or lobed-shaped nuclei containing heterochromatin, as well as lysosomes visible in the cytoplasm. Morphologically, these cells can be identified as microglia (Figure 3(В)). Similar cells were visualized in the SCI group within the extensive posttraumatic cavity.

The wall of the traumatic cavity is formed by several types of cells. The first are astrocytes of the glial scar, which have an elongated shape with electron-dense cytoplasm and an elongated nucleus with highly electron density euchromatin (Figure 3(С)). Such cells may also form bands intersecting the central cavity. In the vicinity of these cells we observed apoptotic bodies, consisting of electron-dense concentric lamellar structures along the periphery (Figure 3(D)). This second cell type had morphology typical of macrophages, also having an elongated shape with electron-dense cytoplasm with lysosomes, and elongated and irregularly shaped nuclei with heterochromatin (Figure 3(С)).

Throughout the tissue there was an increased in the number of blood capillaries in the cavity border (Figure 3(E)), where there was an accumulation of cells with large electron-light nuclei, round or bean-shaped, with small invaginations of karyolemma. This cell type had electron-transparent cytoplasm with electron-dense small mitochondria and processes extending between the nerve elements and vessels. These morphological characteristics indicate that these cells are astrocytes (Figure 3(F)). Amongst them there were a few small cells, microglia, with electron-dense nuclei and cytoplasm in which the cisternae of endoplasmic reticulum had lighter intermembrane spaces (Figure 3(F)).

Rostral to the epicenter of the injury, there were clusters of small myelinated fibers with very thin myelin sheaths (Figure 3(Н)). There were signs of mesaxon myelination on the axial cylinder (Figure 3(G)). Myelin-forming cells were probably oligodendrocytes and Schwann cells, which are often found (Figure 3(J)). Schwann cells have an average electron density cytoplasm with a large irregular or bean-shaped nucleus containing heterochromatin. The basement membrane covers the entire surface of Schwann cells. These cells were also identified in SCI group, but at lower levels, more often in the cells islets in the posttraumatic cavity. Most of the Schwann cells in SCI group contained features of apoptosis and myelin sheath disintegration by day 30 after injury compared to the SCI hUCB-MCs group. Rostral and caudal to the epicenter of the injury amongst the myelinated fibers we observed unmyelinated fibers with indistinct borders, between which intercellular synaptic contacts were formed. In the white matter (lateral and ventral funiculi) in the caudal and rostral direction from the epicenter of the injury, there were clusters of fibrous astrocytes with oval electron-light nuclei containing euchromatin (Figure 3(I)). These cells had cytoplasm with numerous parallel extending bundles of intermediate filaments and highly condensed mitochondria. The form of these astrocytes was indistinct and their processes come between nerve elements, forming long, wide bands.

#### 3.3.2. The Morphological and Ultrastructural Features of the Spinal Cord Tissue at Day 30 after SCI and Transplantation of hUCB-MCs + Ad5-VEGF + Ad5-GDNF

**Figure 4:**
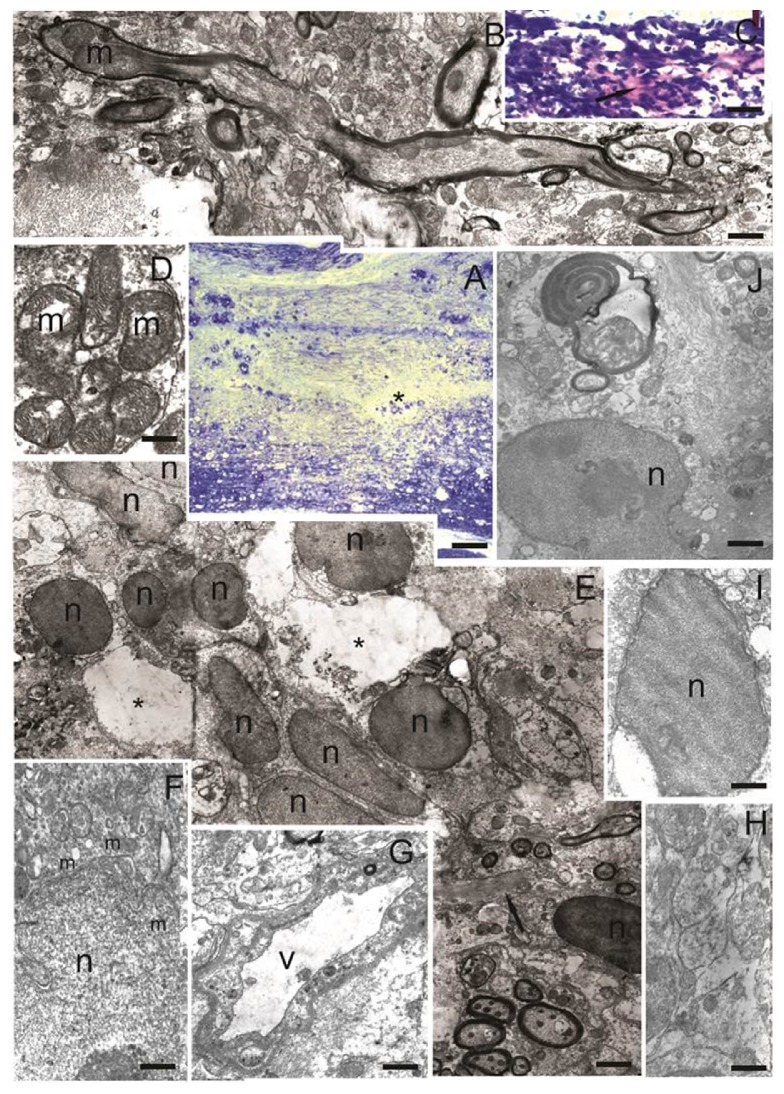
The morphological and ultrastructural features of the spinal cord tissue at day 30 after SCI and transplantation of hUCB-MCs + Ad5-VEGF + Ad5-GDNF. (А) Longitudinal section of the spinal cord (azure-eosin stain). Asterisk shows small posttraumatic cavities. (B) Myelinated fiber with growth cone contained mitochondria, which are greatly enlarged in size. (C) Red-purple precipitate reveals the presence thin bundles of collagen fibers in the epicenter of injury (arrow). (D) Accumulation of mitochondria with clear cristae in a cross section of unmyelinated fibers. (E) Accumulation of two cell types in the area of injury. Asterisks show small posttraumatic cavities. Arrow indicates process of fibrous astrocyte contained bundles of parallel extending intermediate filament. (F) Astrocyte with cytoplasm rich in next organelles: Golgi complex, mitochondria, multivesicular bodies, and greatly enhanced, almost circular, cisterns of rough endoplasmic reticulum. The boundaries of cell are unclear; processes come between the surrounding fibers. (G) Blood capillaries in the area of injury. (H) Unmyelinated nerve fibers in the area of injury. (I) Microglia in posttraumatic cavity. The nucleus occupies most of the cytoplasm and contains euchromatin. (J) Fibrous astrocyte with electron-transparent cytoplasm and electron-light nucleus with irregular shape. Processes of these cells contained bundles of parallel extending intermediate filament and come between regenerating and degenerating nerve elements. Scale bar: 200 *μ*м (А), 5 *μ*м (В, E), 100 *μ*м (С), 500 nm (D, F, H), 1 *μ*м (G), and 3 *μ*м (I, J). m: mitochondria; n: nucleus; v: vessel.

At day 30 after SCI and transplantation of hUCB-MCs + Ad5-VEGF + Ad5-GDNF, we did not observe a major posttraumatic cavity compared to the SCI and SCI hUCB-MCs groups. The whole area of injury epicenter was filled with regenerating nerve elements. We detected only small cavities (Figure 4(A)), in which there were sometimes residues of degenerating cell and fibers. Weigert-Van Gieson's stain revealed the presence thin bundles of collagen fibers in the epicenter of injury (Figure 4(C)), which were also found in the SCI and SCI hUCB-MCs groups. Both along the edge and inside the small cavities we observed single cells which were morphologically similar to microglia (Figure 4(I)). The area of injury was riddled a large number of blood capillaries (Figure 4(G)), amongst which there were many unmyelinated and myelinated nerve fibers (Figure 4(Н)). In the epicenter of the injury we detected intensive formation of axon growth cones (Figure 4(B)). Such structures were not found in the SCI and SCI hUCB-MCs groups. The cytoplasm of axons in the area of the growth cone contained greatly enlarged mitochondria. Such mitochondria had an oval or highly elongated shape with clear cristae and swelling of the matrix with a slight enlightenment in the center often present (Figure 4(D)).

Thirty days after SCI and transplantation of hUCB-MCs + Ad5-VEGF + Ad5-GDNF, the whole injury area contained sprouted bands of two cell types (Figure 4(E)). The first were cells with a narrow rim of cytoplasm around oval or bean-shaped electron-dense nuclei containing euchromatin. These cells were similar in size and morphology to human mononuclear cells. Engrafted cells were localized by the bands along which regenerating fibers and blood capillaries started developing. The second cell type had electron-transparent elongated nuclei with euchromatin. The cytoplasm of these cells was electron-light and contained the following organelles: Golgi complex, mitochondria, multivesicular bodies, and greatly enhanced and almost circular cisterns of rough endoplasmic reticulum (Figure 4(F)). These structural features indicate that these cells actively synthesize products that are destined for the extracellular space. Bands of such cells were solely detected in SCI hUCB-MCs + Ad5-VEGF + Ad5-GDNF group and not in the SCI and SCI hUCB-MCs groups.

Fibrous astrocytes were identified in the epicenter of the injury. The processes of these cells contained bundles of parallel extending intermediate filaments and were located between regenerating and degenerating nerve elements (Figure 4(J)). These astrocytes have a similar structure to the fibrous astrocytes in SCI and SCI hUCB-MCs groups, which were identified in rostral and caudal from the epicenter of the injury.

### 3.4. Immunoelectron Microscopic Analysis Revealed Directions of hUCB-MCs Differentiation in the Area of SCI

GFAP-positive astrocytes with an irregular shape, located on the edge of the large posttraumatic cavity, as well as caudal and rostral from the injury epicenter, were identified in the SCI group (data not shown). As expected HNA was not detected in the SCI group.

**Figure 5:**
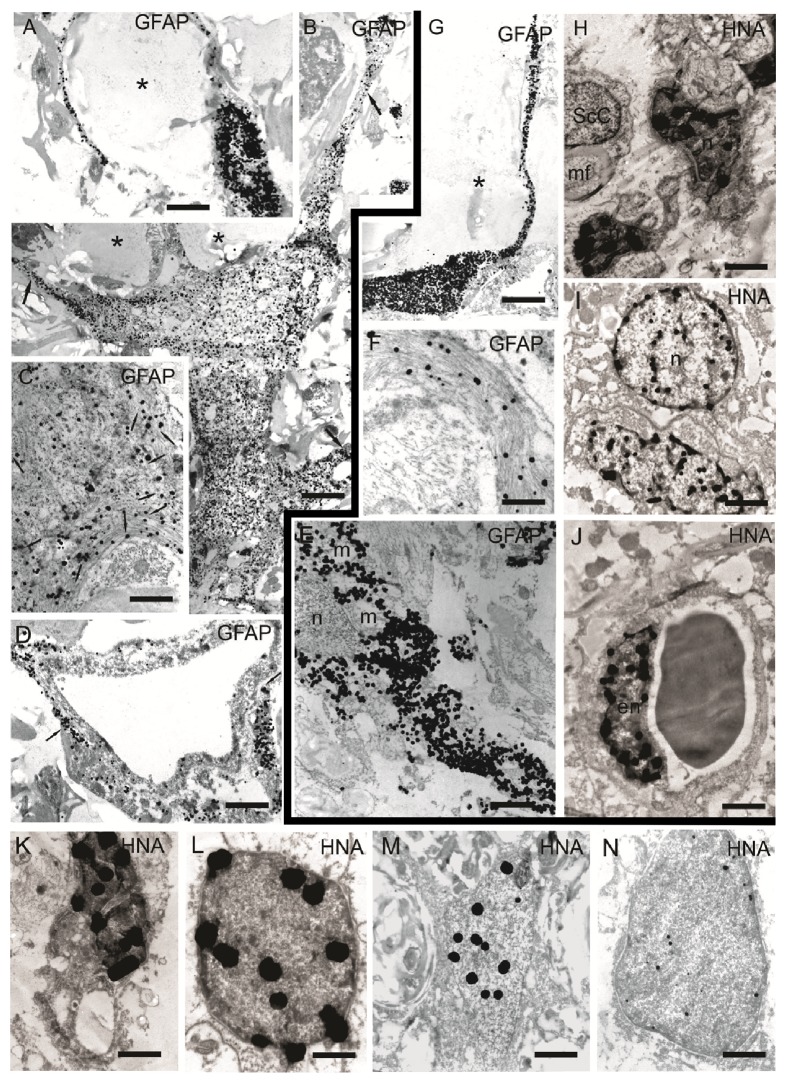
Immunoelectron microscopic results. (А–D), (K–N) Immunohistochemical reaction in the area of spinal cord injury in hUCB-MCs + Ad5-VEGF + Ad5-GDNF group. (A) GFAP-positive astrocyte with narrow processes which limit small posttraumatic cavity (asterisk). (B) GFAP-positive fibrous astrocyte with numerous branched processes (arrows), which surround the cavity (asterisks) and form myelinated and unmyelinated fibers. (С) Cytoplasm of GFAP-positive fibrous astrocyte. Colloidal gold particles sit exclusively on intermediate filaments in the cytoplasm of cells (arrows). (D) GFAP-positive reaction (arrows) around the capillaries. (K) HNA-positive reaction in microglia nucleus. (L) HNA-positive cells with round nucleus and narrow rim of cytoplasm. (M) HNA-positive reaction in fibrous astrocyte nucleus. (N) HNA-positive reaction in elongated cell nucleus in the area of injury. (E–J) Immunohistochemical reaction in the area of spinal cord injury in hUCB-MCs group. (Е) GFAP-positive fibrous astrocyte. (F) GFAP-positive intermediate filaments in fibrous astrocyte cytoplasm. (G) GFAP-positive narrow astrocyte with thin processes. (H) HNA-positive reaction in microglia nucleus. (I) HNA-positive elongated cell with oval nucleus and oval cells with a round nucleus. (J) HNA-positive endothelial cell. Scale bar: 5 *μ*м (A, D, G, J), 1 *μ*м (B, N, E, I), 200 nm (C), 500 nm (K), 300 nm (L, F), 700 nm (M), and 800 nm (H). n: nucleus; mf: myelin fiber; m: mitochondria; en: endothelial cell; ScC: Schwann cell.

In the SCI hUCB-MCs group the walls of the large traumatic cavities were lined with GFAP-positive narrow cells with thin processes (Figure 5(G)), and a large number of fibrous astrocytes with cytoplasm containing GFAP-positive intermediate filaments were also identified (Figures 5(F) and 5(E)). Numerous HNA-positive microglia-like cells, which formed bands in the large posttraumatic cavity (Figure 5(H)), and HNA-positive elongated cells with oval nuclei containing euchromatin, and oval cells with round nuclei were observed in the area of injury in the SCI hUCB-MCs group (Figure 5(I)). Endothelial cells lining the numerous newly formed capillaries in the area of posttraumatic cavity were also HNA-positive (Figure 5(J)).

A GFAP-positive reaction was detected in the cytoplasm of flattened cells and their narrow processes which restrict posttraumatic small cavity in SCI hUCB-MCs + Ad5-VEGF + Ad5-GDNF group (Figure 5(А)). GFAP-positive fibrous astrocytes were also observed in this group (Figure 5(В)). Colloidal gold particles sat exclusively on intermediate filaments in the cytoplasm of these cells (Figure 5(С)). A GFAP-positive reaction was also detected around capillaries (Figure 5(D)). HNA-positive microglia-like cells were present in small cavities in the SCI hUCB-MCs + Ad5-EGFP group (Figure 5(K)). Round cells with a narrow rim of cytoplasm surrounding a round HNA-positive nucleus, containing euchromatin, were identified as undifferentiated engrafted hUCB-MCs (Figure 5(L)). HNA was also found in the nuclei of fibrous astrocytes (Figure 5(M)) and in elongated cells with oval nuclei in the area of injury (Figure 5(N)). No Schwann cells positive for HNA were found in any group (Figure 5(H)).

## 4. Discussion

Numerous studies have been devoted to the use of hUCB-MCs in cell and cell-mediated gene therapy for SCI, with many clinical trials having been conducted [25]. Although there is much data, many questions, particularly of a translational nature, remain unresolved. Our data indicate that just one cell-mediated delivery of a combination of* vegf* and* gdnf* genes into the site of a lesion, immediately following SCI, improves the recovery of spinal cord function.

At day 30 after SCI and transplantation of nontransduced hUCB-MCs, we observed an improvement in the H and M waves (*A*_max_ of M wave, LP of H wave) compared to the no-transplantation group. An improvement in the H and M waves (*A*_max_ of M wave, LP of M and H wave) was also observed for the genetically modified hUCB-MCs. However, on the criterion of ratio between *A*_max_ of H wave and *A*_max_ of M wave (H_max_/M_max_), transplantation of genetically modified hUCB-MCs into the area of SCI was more effective than the injection of native hUCB-MCs. Therefore, it is possible to compare growth in the value of H_max_/M_max_, as the most significant indicator of the H-reflex, with augmentation the total value of the BBB rating scale after cell and gene-cell therapy [23].

Previously, in a similar experimental model using hUCB-MCs + Ad5-VEGF + Ad5-GDNF transplantation, with verified overexpression of VEGF and GDNF mRNA at 30 days after injury, we showed an increase in the preservation of spinal cord tissue in the area of contusion injury, which was more pronounced than in nontransduced hUCB-MC [23]. Using immunoelectron microscopy, we demonstrated that engrafted HNA^+^ cells with the morphology of phagocytes and microglia-like cells formed compact clusters or cell bridges within the traumatic cavities that were lined by GFAP^+^ host astrocytes. The effect of filling posttraumatic cavities with engrafted hUCB-MCs indicates that tissue repair is occurring. This active regeneration and filling of the cavity may be essential for initiating and maintaining axonal growth.

In addition to increases in spared tissue, the numbers of myelinated fibers had increased in the lateral and ventral funiculi at a distance of 2.5 mm from the lesion epicenter by day 30 after SCI and transplantation of hUCB-MCs. In the CST, genetically modified hUCB-MCs significantly increased the number of spared myelinated fibers compared to nontransduced hUCB-MCs (22 fold, *P* > 0.01). The same effect was demonstrated in a similar experimental setup using the hUCB-MCs + Ad5-VEGF + Ad5-GDNF transplantation model, where sparing/regeneration of CGRP^+^ and GAP43^+^ axons correlated with a reduction in reactive astrogliosis and expression of GFAP [23]. The data presented also corroborates the electrophysiology data, showing a decrease in the speed of conduction of the motor fibers compared to the SCI hUCB-MCs + Ad5-VEGF + Ad5-GDNF group.

We observed HNA-positive cells with structures typical of endothelial cells in the lining of capillaries in the injury area. Evidence for grafted cells in the endothelium of capillaries has been shown previously [26], but the data did not indicate that these cells had transmigrated through the vessel wall and differentiated into endothelial cells. Our data indicates differentiation of progenitors in hUCB-MC population into endothelial cells. Furthermore, capillaries containing HNA-positive endothelial cells derived from hUCB-MCs at the site of SCI suggest that angiogenesis is occurring and supports the strategy of using stem cells to deliver genes encoding proangiogenic factors.

One of the possible and massive directions of hUCB-MCs differentiation is the formation of phagocytes. Detection of HNA in the nuclei of these cells indicates that they are derived from engrafted cells. These round cells have round nuclei and all the other characteristic cytoplasmic structures of phagocytic cells. Thirty days after SCI, this was the subpopulation containing the highest number of grafted cells. For these cells, alternative pathways of differentiation could include formation of microglial cells or macrophages. Previously our group reported that hUCB-MCs may differentiate into microglia-like (Iba1^+^) cells in spinal cord after transplantation in ALS mice [26]. Elucidation of the differentiation pathways requires further studies including immunocytochemical typing.

Schwann cells appearing in the area of SCI and involved in transient remyelination in oligodendrocyte deficits conditions have long attracted the attention of researchers. In the SCI area, we observed cells, identified as Schwann cells by morphological criteria such as a thickened myelin sheath, the presence of a typical basement membrane, and accumulation of collagen fibers near the contact surface. These Schwann cells did not express HNA, indicating an endogenous origin, suggesting that they either migrate from peripheral nerve structures to the damaged area or are resident glial progenitors (CNS-resident glial progenitor/stem cells) [27]. Further research is needed to evaluate the potential impact of hUCB-MCs transplantation and cell-mediated gene delivery of* vegf* and* gdnf* on the population of Schwann cells to determine their origins and capacity for remyelination after SCI.

Assessment of the effectiveness of* vegf* and* gdnf* gene delivery (individually and combined) by means of hUCB-MCs in spinal cord regeneration requires a comparative analysis. Thus far, we have shown that transplantation of hUCB-MCs, transduced with Ad5-GDNF, into the area of SCI damage improves tissue preservation, reduces the formation of cavities, and inhibits degeneration of myelinated fibers [8, 28]. It is unclear, however, how transplantation of hUCB-MCs transduced with Ad5-VEGF alone functions in the same experimental model of SCI. Therefore, it will be important to determine the contribution of each transgene expressed individually, as well as potential synergistic actions after simultaneous delivery of both* vegf* and* gdnf* genes in hUCB-MCs transduced with adenovirus vectors.

Our results showed that hUCB-MCs transplanted into the site of SCI improved regeneration, with hUCB-MCs genetically modified with the VEGF and GNDF genes being the most effective.
